# Ultra-early Spinal Decompression Surgery Can Improve Neurological Outcome of Complete Cervical Spinal Cord Injury; a Systematic Review and Meta-analysis

**DOI:** 10.22037/aaem.v10i1.1471

**Published:** 2022-01-31

**Authors:** Mahmoud Yousefifard, Behrooz Hashemi, Mohammad Mehdi Forouzanfar, Rozita Khatamian Oskooi, Arian Madani Neishaboori, Reza Jalili Khoshnoud

**Affiliations:** 1 Physiology Research Center, Iran University of Medical Sciences, Tehran, Iran.; 2 Research Center for Trauma in Police Operations, Directorate of Health, Rescue and Treatment, Police Force, Tehran, Iran.; 3 Men’s Health and Reproductive Health Research Center, Shahid Beheshti University of Medical Sciences, Tehran, Iran.; 4 Emergency Department, Imam Reza Hospital, Birjand University of Medical Sciences, Birjand, Iran.; 5 Functional Neurosurgery Research Center, Shohadaye Tajrish Comprehensive Neurosurgical Center of Excellence, Shahid Beheshti University of Medical Sciences, Tehran, Iran.

**Keywords:** Decompression, Surgical, Spinal Cord injuries, Neurological Rehabilitation

## Abstract

**Introduction:**

Early decompression within the first 24 hours after spinal cord injury (SCI) is proposed in current guidelines. However, the possible benefits of earlier decompression are unclear. Thus, the present meta-analysis aims to investigate the existing evidence regarding the efficacy of ultra-early decompression surgery (within 12 hours after SCI) in improving patients’ neurological status.

**Methods:**

A search was performed in Medline, Embase, Scopus and Web of Science electronic databases, until the end of August 2021. Cohort studies and clinical trials were included in the present study. Exclusion criteria were absence of an early or late surgery group, failure to report neurological status based on the American spinal injury association impairment scale (AIS) grade, failure to perform the surgery within the first 12 hours after SCI, and duplicate reports and review articles. Two independent reviewers performed data collection, and risk of bias and certainty of evidence assessments. The outcome was reported as odds ratio (OR) and 95% confidence interval (CI).

**Results:**

Data from 16 articles, which studied 868 patients, were included. Compared to early or late decompression surgery, ultra-early decompression surgery significantly improves patients’ neurological status (OR = 2.25; 95% CI: 1.41 to 3.58). However, ultra-early surgery in thoracolumbar injuries is not significantly more effective than early to late surgery. Moreover, ultra-early surgery in patients with a baseline AIS A increases the chance of neurologic resolvent up to 3.86 folds (OR=3.86; 95% CI: 1.50 to 9.91). Contrastingly, ultra-early surgery does not result in significant improvement compared to early to late surgery in patients with AIS B (OR = 1.32; 95% CI: 0.51 to 3.45), AIS C (OR = 1.83; 95% CI: 0.72 to 4.64), and AIS D (OR = 0.99; 95% CI: 0.31 to 3.17).

**Conclusion:**

Current guidelines emphasize that spinal decompression should be performed within 24 hours after SCI, regardless of injury severity and location. However, results of the present study demonstrated that certain considerations may be taken into account when performing decompression surgery: 1) in patients with AIS A injury, decompression surgery should be performed as soon as possible, since its efficacy in neurological improvement is 3.86 folds higher in the first 12 hours after injury. 2) ultra-early decompression surgery in patients with cervical injury is more effective than in patients with thoracic or lumbar injuries. 3) postponing decompression surgery to 24 hours in SCI patients with AIS B to D does not significantly affect the neurological outcome.

## 1. Introduction:

Spinal cord injuries (SCIs) are one of the most debilitating traumas, for which there is no definitive cure. Furthermore, the risk of premature death in SCI patients is five times higher than that of healthy people. Statistics show that in 2016, about one million people suffered from SCI. as a result, SCI is responsible for a considerable proportion of the burden of trauma accidents (1).

Current therapies for SCI are surgical interventions and pharmacological treatments. Spinal decompression surgery is the most important surgical intervention within the first 24 hours after the injury (2).

Current guidelines demonstrate that surgical decompression within the first 24 hours improves six-month outcome in the patients, regardless of the level of SCI (2). Although these guidelines emphasize performing surgical decompression within the first 24 hours, the efficacy of ultra-early decompression surgery within the first 12 hours after SCI is yet to be determined. In this regard, in 2020, a meta-analysis showed that ultra-early decompression surgery can result in significant neurological improvement in SCI patients. However, the meta-analysis did not consider the severity and the location of the injury (3). Another meta-analysis depicted that ultra-early surgery within the first 8 hours, improves neurological status of the patients with complete SCI, but it is not significantly effective in patients with partial injury (4). Moreover, a meta-analysis performed in 2016 demonstrated that surgical decompression in the first 12 hours after injury may lead to a neurological improvement of up to 74%, while surgical decompression in the first 24 hours results in a neurological improvement of up to 25% (5).

Nevertheless, ultra-early decompression surgery following SCI encounters a number of challenges. As one of the most important limitations, performing the surgery within the first 8 to 12 hours is usually impossible, because of the delay in transporting the patients to medical facilities and preparing them for the surgery. However, if enough evidence supports the superiority of ultra-early decompression surgery to early decompression injury, in terms of efficacy in neurological improvement, current guidelines may be reconsidered. Yet, lack of consensus over the matter withholds a general conclusion regarding the efficacy of ultra-early decompression surgery in SCI patients. As a result, the present meta-analysis gathered existing evidence comparing the efficacy of ultra-early surgery (within 12 hours) with early (within 24 hours) and late (after 24 hours) decompression surgery. Moreover, the efficacy of ultra-early surgery in different severities of SCI was also assessed.

## 2. Method:

**2.1 Study design**

The present study is a systematic review and meta-analysis designed based on the PRISMA 2020 guidelines, performed to gather the existing evidence regarding the efficacy of ultra-early surgery (within the first 12 hours after SCI) in humans. The protocol of the present study has not been previously registered and published in its current form.

**2.2 Eligibility criteria**

PICO, in the current study, was defined as patients with traumatic SCI (P or patients) who underwent ultra-early decompression surgery (I or intervention). Comparison (C) was made with early (surgery within 24 hours) and late (surgery after 24 hours) decompression surgery performed on SCI patients. Finally, the evaluated outcome (O) was neurological improvement based on the American Spinal Injury Association (ASIA) impairment scale (AIS) grade. Therefore, cohort and clinical trials were selected to enter the present study. Furthermore, exclusion criteria were lack of an early or late surgery group, failure to define an exact cut-off time point as the definition of ultra-early, failure to report the neurological status of the patients based on the AIS grades, failure to perform the surgery within the first 12 hours after the injury, duplicate reports, and review articles.

**2.3 Search strategy**

The systematic search was performed in Medline, Embase, Scopus, and Web of Science electronic databases until the end of August 2021. Search strategy and the selected keywords for Medline database are depicted in Appendix 1. In addition to the systematic search, a manual search was conducted in google and google scholar search engines. Moreover, references of the related articles were screened to find additional articles.

**2.4 Data collection**

Two independent reviewers screened titles and abstracts of the articles and gathered full texts of the related ones. Then, the gathered full texts were thoroughly studied and completely related articles were selected. Afterwards, each of the reviewers extracted required data using a checklist on the Excel program. The data included name of the first author, date of publication, country, study type and design, definition of ultra-early group, definition of late surgery group, location of injury, severity of injury based on AIS grade, definition of neurological improvement, age and gender distribution of samples, number of neurological improvements in ultra-early and late surgery, number of not-improved patients, and follow-up duration. Any disagreements were resolved through discussion with a third reviewer.

**2.5 Quality assessment of included papers**

Since the included studies were either cohort or clinical trials, the quality assessment of the studies were performed based on the guidelines of the National Heart, Lung, and Blood institute (NHLBI) risk of bias assessment tool (6). Moreover, risk of bias of the clinical trials was assessed using Cochrane guidelines (7). Scoring and assessing the risk of bias based on the NHLBI guidelines have been previously discussed (8).

**2.6 Evaluated outcomes**

The evaluated outcome was improvement of the neurological status of the patients following surgical decompression based on the AIS grades. This scale is a five-point scoring scale, covering complete sensory-motor injury (AIS A) to normal status (AIS E). The reason for selecting AIS scoring system was that even one point improvement based on this scale is considered as a clinically significant improvement and a sign of decrease in inflammation (9).

2.7 Certainty of evidence

The level of evidence was scored based on the Grading of Recommendations, Assessment, Development and Evaluations (GRADE) criteria (10). The level of evidence of each study was scored by two independent researchers based on the existence of risk of bias, imprecision, indirectness, inconsistency, and publication bias.

**2.8 Data synthesis and statistical analysis**

The cut-off point used to define the ultra-early surgery varied between four to 12 hours after the injury. Since most of the previous articles defined ultra-early as eight or 12 hours after the injury, the 12 hours cut-off point was adopted as the definition of ultra-early surgery in the present study. Data were recorded as number of improved and non-improved cases in ultra-early (surgery within 12 hours), early (surgery within 24 hours), and late (surgery after 24 hours) groups. Some studies reported the outcome of surgery after 12 hours as a whole and did not discriminate early and late surgeries. Therefore, the efficacy of ultra-early decompression surgery in promoting neurological recovery was compared with early to late surgery in a pooled analysis. Furthermore, data were recorded with respect to AIS grades.

Data were entered to STATA 17.0 statistical program, and the analyses were performed using Meta command. Since the study design and the evaluated SCI severity differed between the studies, researchers in the present study adopted the random effect model analysis. Heterogeneity and publication bias were assessed using I^2^ test and Egger’s test, respectively.

The efficacy of ultra-early decompression surgery in promoting neurological recovery was reported as a pooled odds ratio (OR) and 95% confidence interval (CI). In addition, subgroup analyses were performed based on the injury location, injury severity, and the cut-off time point for each (early surgery and late surgery). Since age and follow-up time variations were possible confounding factors, a meta-regression was performed to evaluate the possible effects of these confounding factors.

## 3. Results:

**3.1 Study flow and characteristics of included studies**

**Figure 1:**
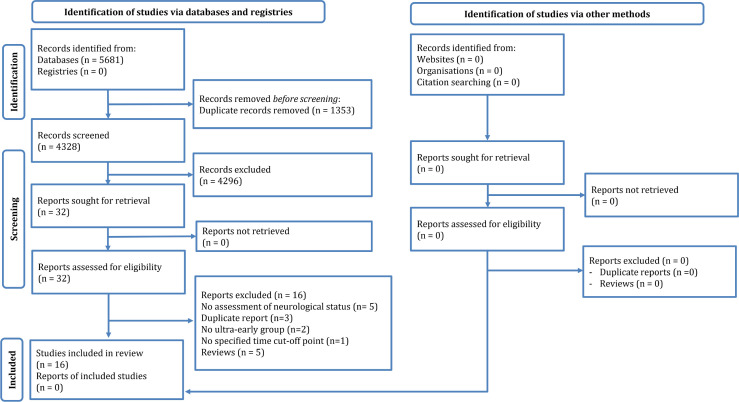
PRISMA flow diagram of the current review

The systematic search yielded 4328 non-duplicate records. After reviewing the articles, 32 possibly related articles were selected, among which 16 studies were included in the present systematic review and meta-analysis (11-26). The reasons for exclusion were failure to evaluate neurological status based on the AIS/Frankel grade (n=5), duplicate reports (n=3), lack of an ultra-early surgery group, failure to define a distinct cut-off point for ultra-early surgery (n=1), and review articles (n=5) (Figure 1).

12 articles were retrospective cohorts, three studies were prospective cohort, and one study was clinical trial. The studies included data from 868 patients (429 patients in ultra-early surgery group and 439 patients in early to late surgery group). Injury location was cervical in 12 studies, thoracolumbar in two studies, and all areas of the spinal cord in two other studies. Injury severity based on the AIS grades were between A to D. Ultra-early was defined as within eight hours after SCI in eight studies and within 12 hours after SCI in five other studies. A cut-off point of four, five, and six hours after the SCI was defined as the definition of ultra-early in one study each. Early surgery (within 24 hours) was reported in five studies, and the other studies combined the data from early and late surgery and the comparison was made between the numbers from ultra-early surgery and that of the early and late surgeries, combined. Follow-up period varied between six (in nine studies) to 68 months. Recovery was defined as at least one grade improvement based on the AIS score. Table 1 shows the included data in the present study.

**3.2 Effect of ultra-early decompression injury on neurological status of SCI patients**

**Figure 2:**
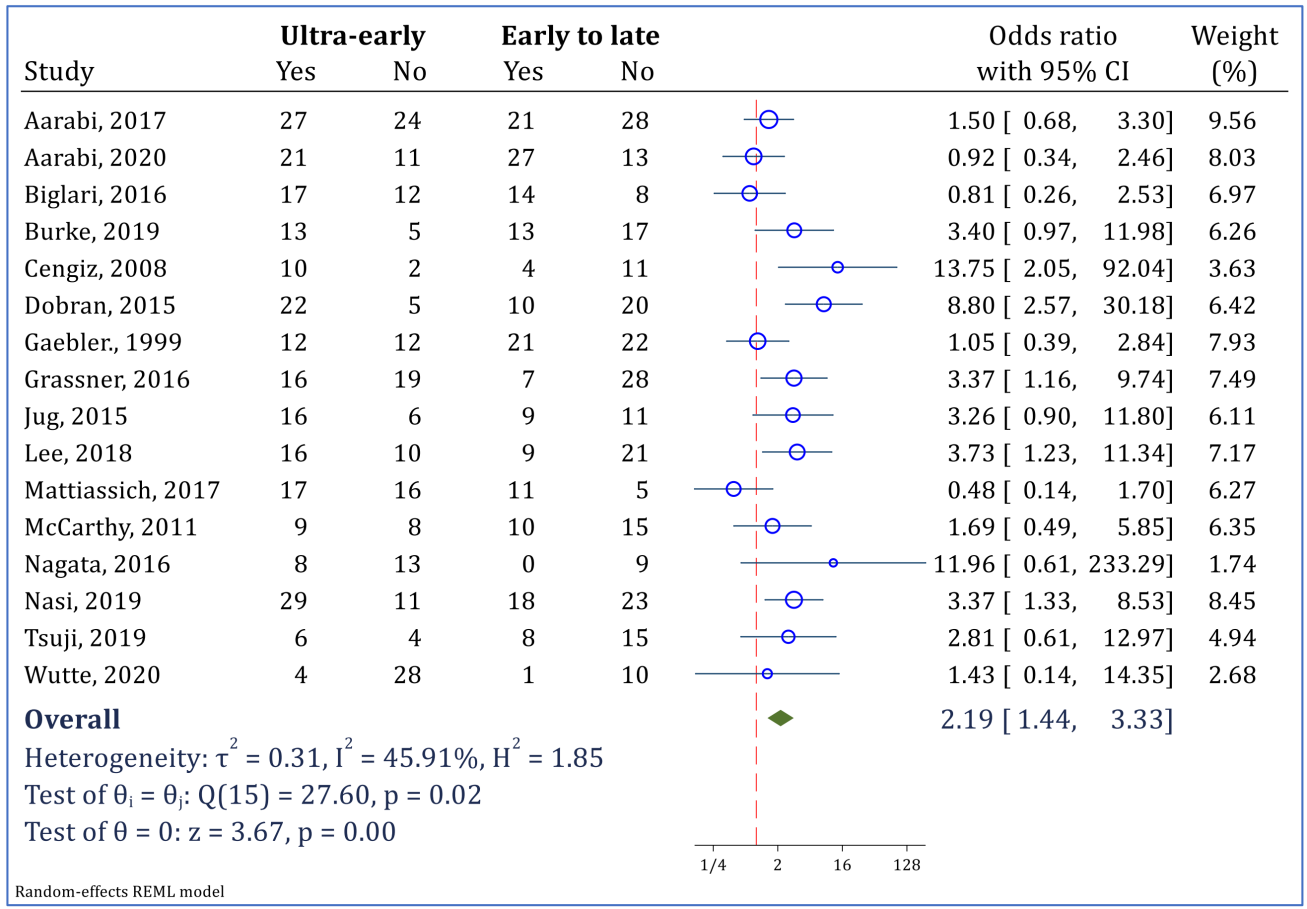
Overall pooled analysis for assessment of ultra-early surgery (< 12 hours) versus early to late surgery (>12 hours) in improvement of neurological status. Neurological improvement was defined as at least 1 grade improvement in American Spinal Injury Association impairment scale (AIS) grade

Overall pooled analysis, regardless of the injury severity, injury location, and the group selected for comparison (early or late surgery) demonstrated that the chances of recovery in patients undergoing ultra-early surgery was 2.19 times higher than that of patients undergoing decompression surgery after 12 hours after injury (OR = 2.19; 95% CI: 1.44, 3.33; I2 = 49.51%) (Figure 2).

**Figure 3:**
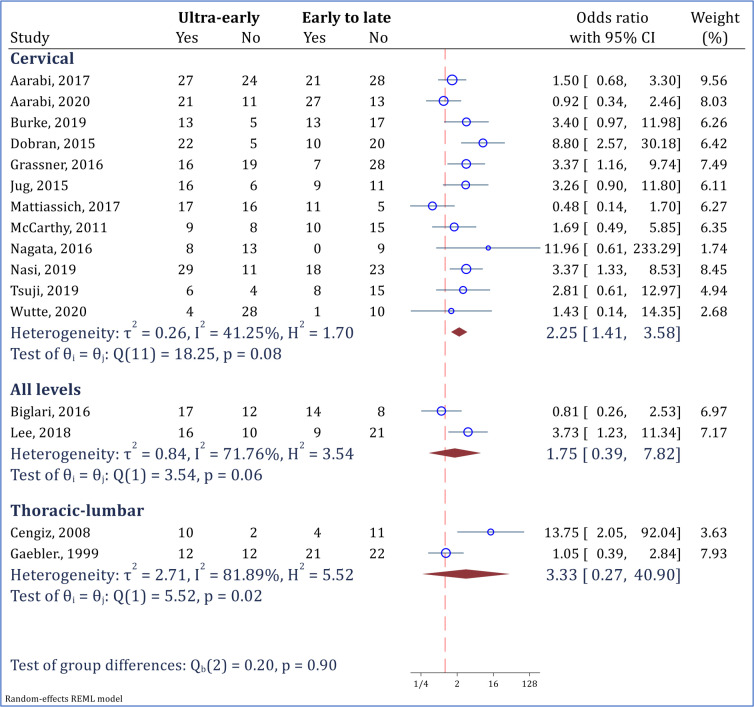
Subgroup analysis for assessment of ultra-early surgery (< 12 hours) versus early to late surgery (>12 hours) in improvement of neurological status based on the level of injury. Neurological improvement was defined as at least 1 grade improvement in American Spinal Injury Association impairment scale (AIS) grade

Subgroup analysis demonstrated that ultra-early surgery was significantly more effective than early to late surgery in improving neurological status of the patients experiencing a cervical injury (OR = 2.25; 95% CI: 1.41 to 3.58; I2 = 41.25%). However, ultra-early surgery did not show a significantly superior efficacy in thoracolumbar injuries, in comparison with early to late surgeries (Figure 3).

**Figure 4:**
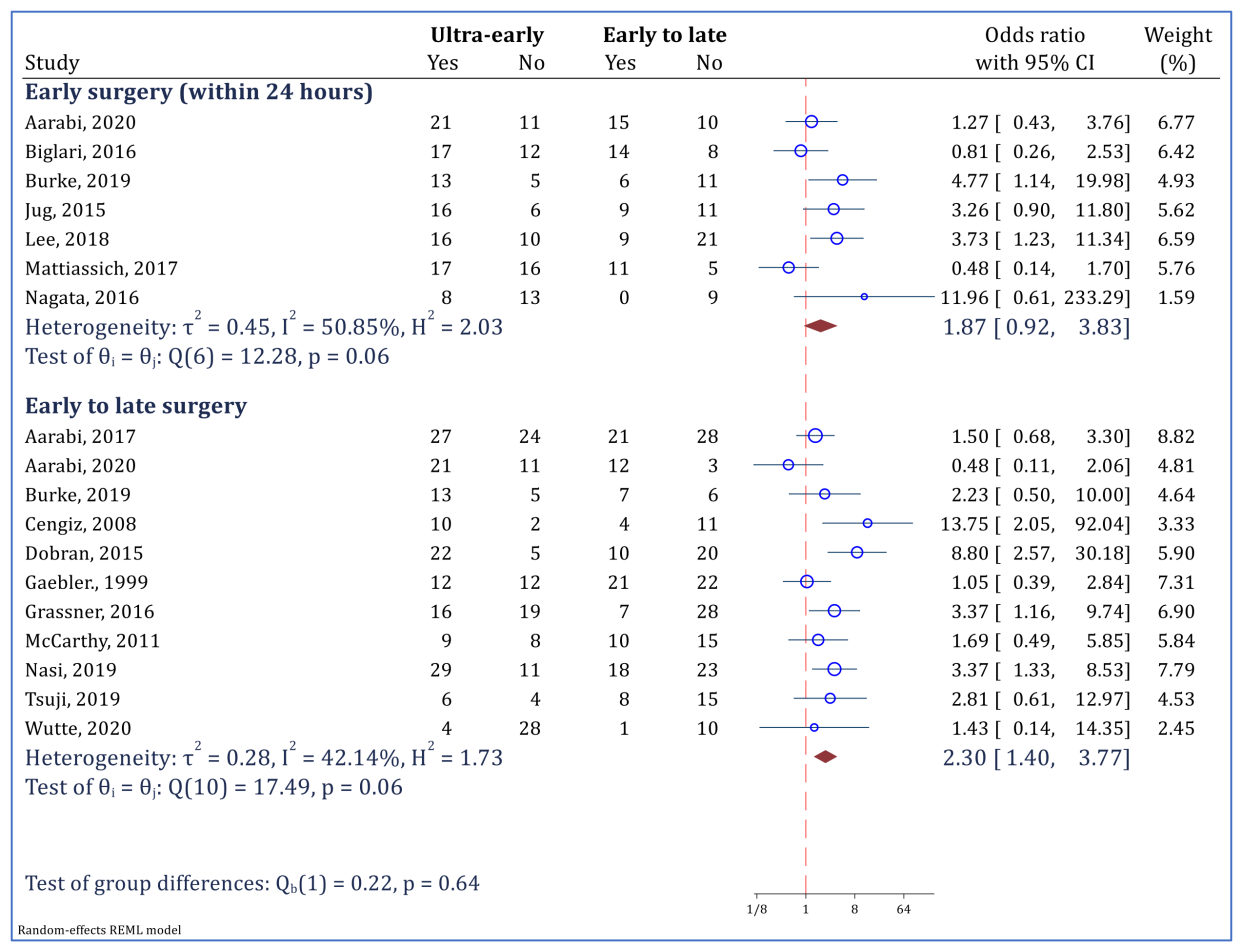
comparison of ultra-early surgery (< 12 hours) and early to late surgery (including surgery within 24 hours and afterwards) in improvement of neurological status. Neurological improvement was defined as at least 1 grade improvement in American Spinal Injury Association impairment scale (AIS) grade

It is noteworthy that ultra-early surgery is not more effective than early surgery (within 24 hours after SCI), and the observed neurological status recovery was similar in both surgeries. (OR = 1.87; 95% CI: 0.92 to 3.83; I2 = 50.85%). However, the observed efficacy of ultra-early surgery in neurological recovery was significantly higher than that of early to late surgery (OR = 2.30; 95% CI: 1.40 to 3.77; I2 = 42.14%) (Figure 4). 

**3.3 Effect of ultra-early decompression surgery on neurological status based on baseline AIS score**

**Figure 5:**
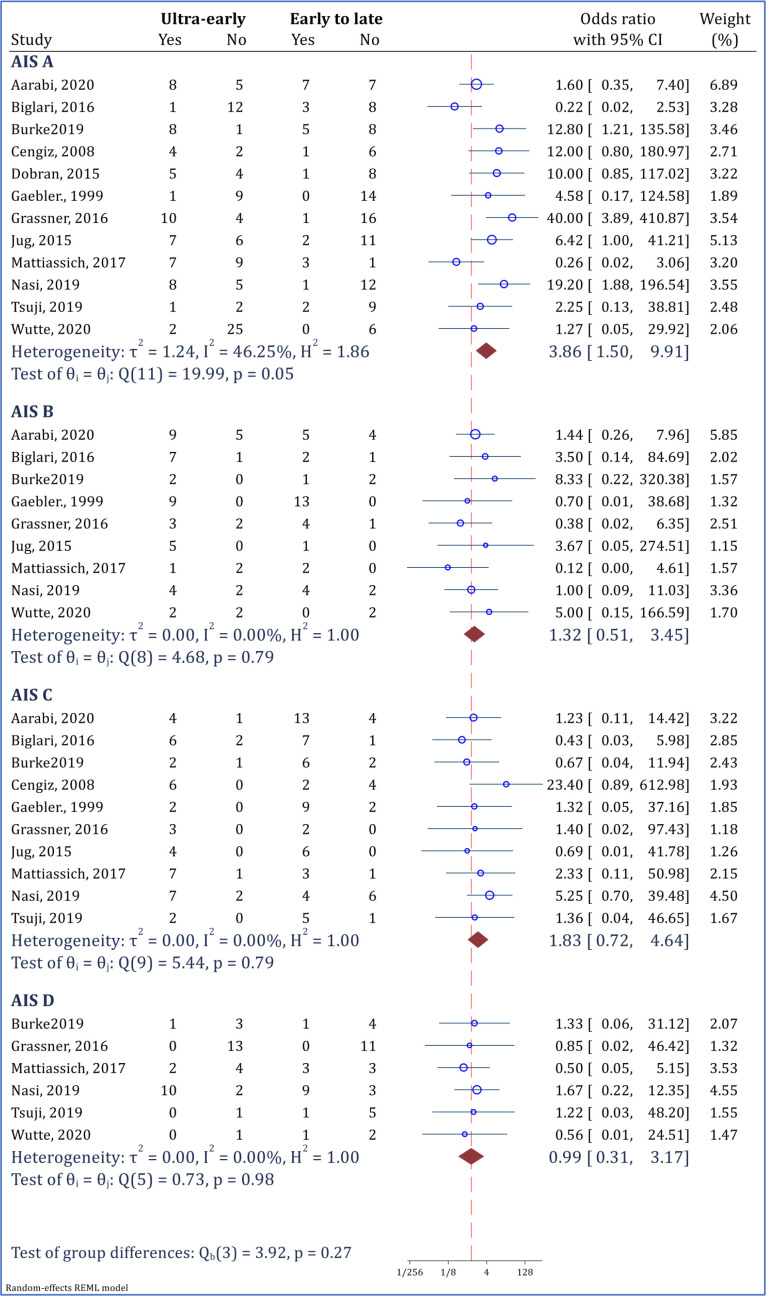
Effectiveness of ultra-early surgery (< 12 hours) in improvement of neurological status based on baseline American Spinal Injury Association impairment scale (AIS) grade. Neurological improvement was defined as at least 1 grade improvement in AIS grade

As a sensitivity analysis, the efficacy of ultra-early decompression surgery based on the baseline AIS score was evaluated. The results demonstrated that ultra-early surgery in patients with a baseline AIS of A can increase the chances of neurological recovery up to 3.86 times (OR = 3.86; 95% CI: 1.50 to 9.91; I2 = 46.25%). Nevertheless, ultra-early surgery was not significantly more effective than early to late surgery in patients with AIS B (OR = 1.32; 95% CI: 0.51 to 3.45; I2 = 0.00%), AIS C (OR = 1.83; 95% CI: 0.72 to 4.64; I2 = 0.00%), and AIS D (OR = 0.99; 95% CI: 0.31 to 3.17; I2 = 0.00%) (Figure 5).

**3.4 Meta-regression**

**Figure 6:**
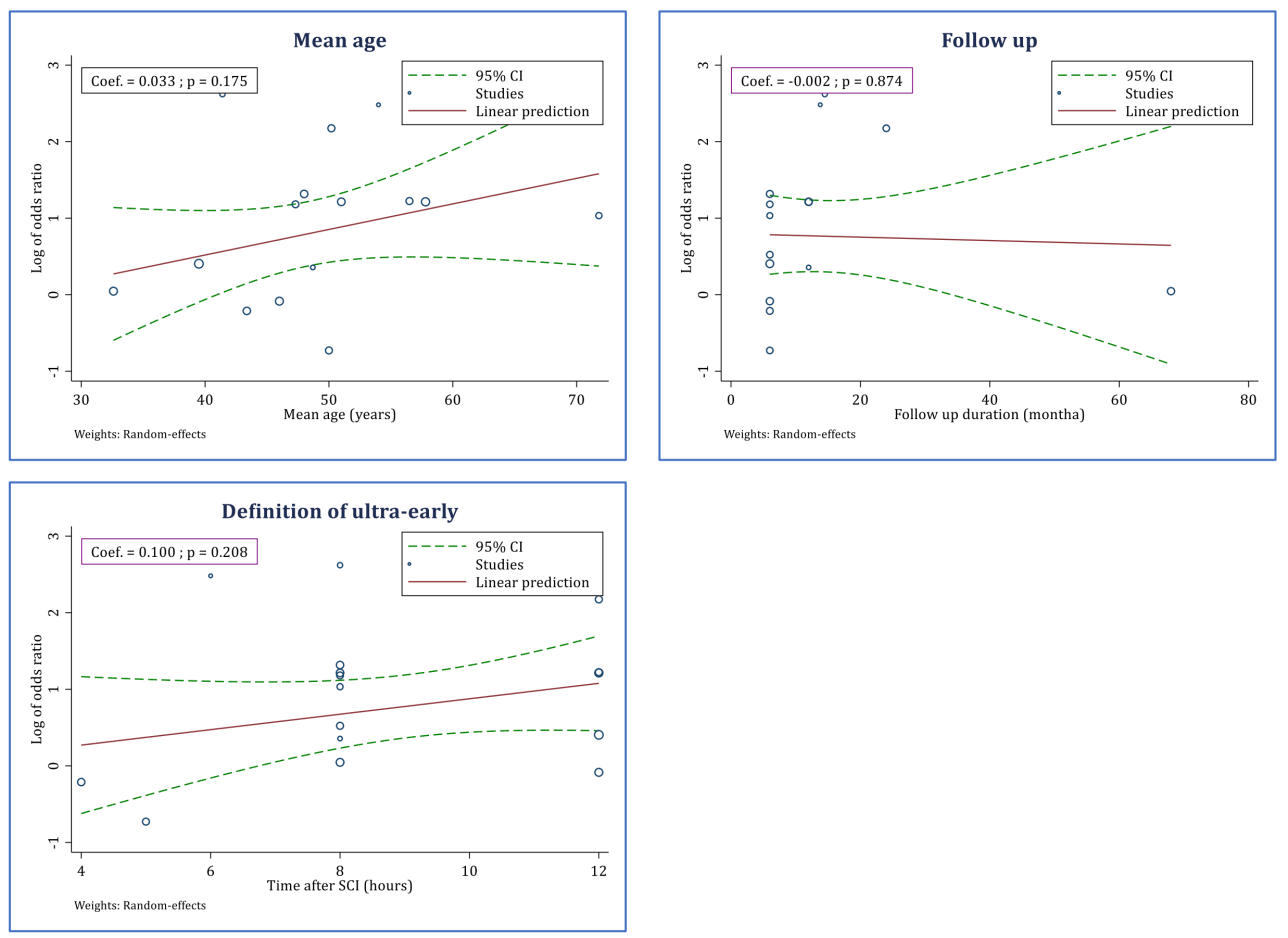
Effectiveness of ultra-early surgery (< 12 hours) in improvement of neurological status based on mean age of patients, follow-up duration, and timing of surgery

Since the mean age and follow-up period were diverse among the studies, a meta-regression was performed with respect to the two variables. The meta-regression demonstrated that the mean age of patients at the time of SCI (meta-regression coefficient = 0.033; p = 0.175) and follow-up time (meta-regression coefficient = -0.002; p = 0.874) were not associated with the efficacy of ultra-early decompression surgery in promoting neurological recovery. Moreover, the meta-regression depicted that ultra-early decompression surgery within the first 12 hours is as effective as when it is performed earlier (meta-regression coefficient = 0.100; p = 0.208) (Figure 6).

**3.5 Risk of bias assessment**

15 cohorts and one clinical trial were included in the present study. Risk of bias of the one clinical trial was low. Moreover, risk of bias assessment of the cohort studies based on NHLBI tool showed that in terms of sufficiency of time frame, the status could not be determined in two studies (signaling question 7). Furthermore, judgment regarding rate of loss to follow-up was not possible in five studies (signaling question 13). Based on the presence of these two fatal errors among the studies, the overall risk of bias in the five studies was scored as high. Also, some risk of bias was found in eight studies, since blinding status and sample size justification were not provided. Risk of bias was considered low in two studies (Table 2).

**3.6 Publication bias**

**Figure 7:**
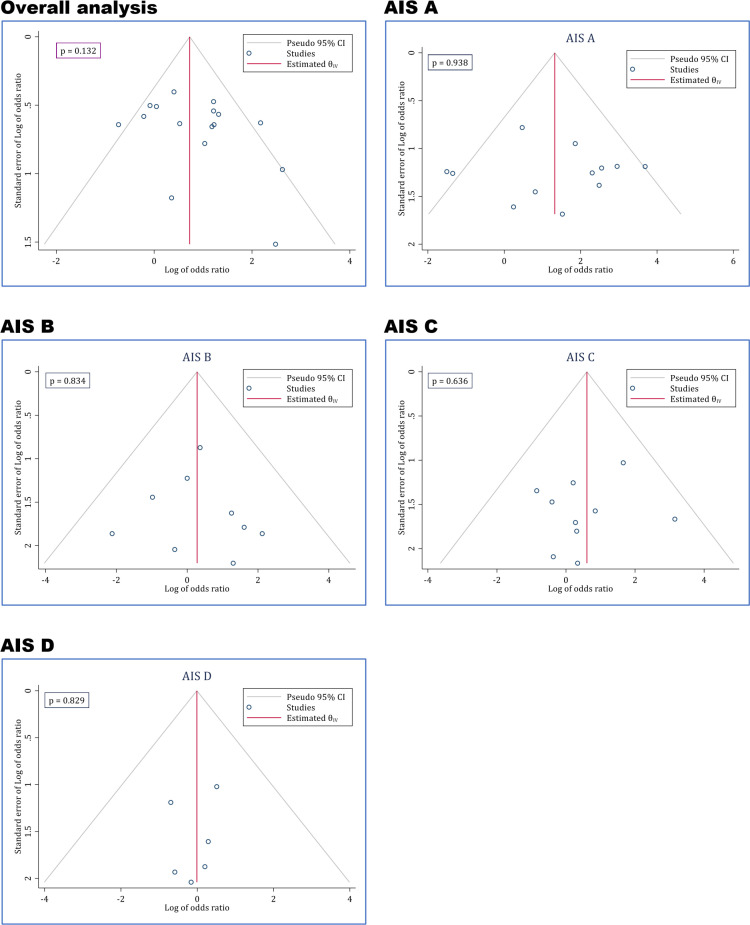
Publication bias assessment of included studies based on AIS grade. AIS: American Spinal Injury Association (ASIA) impairment scale grade

Egger’s test showed no evidence of publication bias among the studies. As depicted in Figure 7, no publication bias existed in the evaluation of the efficacy of ultra-early decompression in improvement of neurological status overall and in various AIS grades.

**3.7 Certainty of evidence**

According to the GRADE’s protocol, the overall level of evidence is low in observational studies. Since there is a possible risk of bias in the present study, one point was deducted from the GRADE score in all outcomes. However, due to the existence of a large magnitude of effect and possible plausible confounders, which increase the confidence of findings, the overall level of evidence was increased and became moderate. Table 3 provides details of our judgments regarding the certainty of evidence.

## 4. Discussion:

Moderate level of evidence demonstrated that ultra-early decompression surgery within the first 12 hours after SCI may improve neurological recovery in patients with cervical SCI with an AIS grade of A. The analyses showed that in comparison with early to late surgery, the ultra-early surgery does not result in a significantly higher efficacy in other severities of SCI (AIS B to D).

Although conflicting results were reported in different studies regarding the prognostic effects of baseline AIS score on the treatment success of spinal decompression surgery (27-30), results of the present study showed that in comparison with early and late surgery, ultra-early surgical decompression can promote neurological recovery in AIS A patients. In these patients, whom are encountered with complete sensory-motor injury, the spinal cord is experiencing devastating pressure. As result, promptly eliminating this pressure may prevent further secondary injuries responsible for a considerable amount of damage following SCI. Hence, this may be the reason behind the observed efficacy of ultra-early early surgery only in AIS A patients. Accordingly, researchers of the present study recommend that ultra-early decompression surgery be performed as soon as possible in patients with complete sensory-motor injury.

Current treatment guidelines emphasize that decompression injury be performed within the first 24 hours, regardless of the injury severity and location. However, current evidences suggest that when performing decompression surgery, certain consideration may be taken into account. These considerations include: 1) in patients with AIS A injury, decompression injury may/should be performed as soon as possible, as it can enhance the chance of neurological recovery to up to 3.86 times. 2) ultra-early decompression surgery is more effective in cervical SCI patients compared to thoracic and lumbar SCI patients. 3) delaying decompression surgery until 24 hours in patients with AIS B to D SCI does not significantly affect the final neurological outcome. 4) different cut-off points were defined for ultra-early decompression injury among the studies, ranging from four to 12 hours. Meta-regression demonstrated that performing ultra-early decompression surgery within a time frame of 12 hours after the injury is as effective as when performed within sooner time points. Therefore, a 12-hour time window after SCI maybe considered as an appropriate definition of performing ultra-early surgery. This definition can promote the possibility of performing ultra-early surgery.

One of the limitations of the present study was the small number of the articles evaluating the results of decompression surgery in thoracic and lumbar spine injuries. Only two studies researched the effects of ultra-early decompression surgery in these patients, which may deduct the power of the performed analyses. As a result, it is recommended that future studies be performed evaluating the effects of ultra-early decompression surgery in patients with lumbar and thoracic SCIs. another limitation of the current study was that only one clinical trial was included. In addition, due to the observational nature of the included studies and the analyses being uncontrolled for possible confounding factors, further clinical trials are encouraged to be performed to overcome the existing limitations.

**Table 1:** Summary of included papers

Study	Design	Ultra-early definition*	Late definition*	Injury location	Follow-up (months)	Severity	Score	Improvement definition	Mean age (years)	No. of males	No. of ultra-early	No. of late
Aarabi, 2017; USA	R-C	12	>12	Cervical	6	A-C	AIS	at least 1 grade	39.5	89	51	49
Aarabi, 2020; USA	R-C	12	12-24; >24	Cervical	6	A-C	AIS	at least 1 grade	46	60	32	40
Biglari, 2016; Germany	P-C	4	4 to 24	All levels	6	A-C	AIS	at least 1 grade	43.37	40	29	22
Burke, 2019; USA	R-C	12	12-24; >24	Cervical	NR	A-C	AIS	at least 1 grade	56.5	38	18	30
Cengiz, 2008; Turkey	RCT	8	>72	Th2-L2	14.5	A-C	AIS	at least 1 grade	41.4	18	12	15
Dobran, 2015; Italy	P-C	12	12 to 72	Cervical	24	A-D	AIS	at least 1 grade	50.2	44	27	30
Gaebler., 1999; Austria	R-C	8	>8	Thoracic-lumbar	68	A-D	Frankel	at least 1 grade	32.6	56	24	43
Grassner, 2016; Germany	R-C	8	>8	Cervical	12	A-D	AIS	at least 1 grade	51	59	35	35
Jug, 2015; Slovenia	P-C	8	8 to 24	Cervical	6	A-C	AIS	at least 1 grade	47.3	34	22	20
Lee, 2018; Korea	R-C	8	8 to 24	All levels	6	A-C	AIS	at least 1 grade	48	35	26	30
Mattiassich, 2017; Austria	R-C	5	5 to 24	Cervical	6	A-D	AIS	at least 1 grade	50	38	33	16
McCarthy, 2011; Australia	R-C	8	>8	Cervical	6	A-D	AIS	at least 1 grade	NR	31	17	25
Nagata, 2016; Japan	R-C	6	6 to 11	Cervical	13.8	A-B	AIS	at least 1 grade	54	28	21	9
Nasi, 2019; Italy	R-C	12	12 to 48	Cervical	12	A-D	AIS	at least 1 grade	57.8	58	40	41
Tsuji, 2019; Japan	R-C	8	>8	Cervical	6	A-D	AIS	at least 1 grade	71.8	35	10	23
Wutte, 2020; Germany	R-C	8	>8	Cervical	12	A-D	AIS	at least 1 grade	48.7	34	32	11

NR: Not reported; P-C: Prospective cohort; RCT: Randomized clinical trial; R-C: Retrospective cohort; AIS: American Spinal Injury Association impairment scale.

*, hours after spinal cord injury.

**Table 2:** Risk of bias assessment of included studies

**Study**	**Q1**	**Q2**	Q3*	**Q4**	**Q5**	Q6*	Q7*	**Q8**	**Q9**	**Q10**	Q11*	**Q12**	Q13*	**Q14**	**Overall**
**Cohort studies according to NHLBI tool**															
Arabi, 2017	Yes	Yes	Yes	Yes	NR	Yes	Yes	No	Yes	NA	Yes	NR	CD	NA	High risk
Arabi, 2020	Yes	Yes	Yes	Yes	NR	Yes	Yes	Yes	Yes	NA	Yes	NR	CD	NA	High risk
Biglari, 2016	Yes	Yes	Yes	Yes	NR	Yes	Yes	Yes	Yes	NA	Yes	NR	CD	NA	High risk
Burke, 2019	Yes	Yes	Yes	Yes	NR	Yes	CD	Yes	Yes	NA	Yes	NR	CD	NA	High risk
Dobran, 2015	Yes	Yes	Yes	Yes	NR	Yes	Yes	No	Yes	NA	Yes	NR	Yes	NA	Some concern
Gaebler., 1999	Yes	Yes	Yes	Yes	NR	Yes	Yes	Yes	Yes	NA	Yes	NR	Yes	NA	Some concern
Grassner, 2016	Yes	Yes	Yes	Yes	NR	Yes	Yes	Yes	Yes	NA	Yes	NR	Yes	NA	Some concern
Jug, 2015	Yes	Yes	Yes	Yes	NR	Yes	Yes	Yes	Yes	NA	Yes	Yes	Yes	NA	Low risk
Lee, 2018	Yes	Yes	Yes	Yes	NR	Yes	Yes	No	Yes	NA	Yes	NR	Yes	NA	Some concern
Mattiassich, 2017	Yes	Yes	Yes	Yes	NR	Yes	Yes	Yes	Yes	NA	Yes	NR	Yes	NA	Some concern
McCarthy, 2011	Yes	Yes	Yes	Yes	NR	Yes	Yes	No	Yes	NA	Yes	NR	Yes	NA	Some concern
Nagata, 2016	Yes	Yes	Yes	Yes	NR	Yes	Yes	No	Yes	NA	Yes	NR	CD	NA	High risk
Nasi, 2019	Yes	Yes	Yes	Yes	NR	Yes	Yes	Yes	Yes	NA	Yes	NR	Yes	NA	Some concern
Tsuji, 2019	Yes	Yes	Yes	Yes	NR	Yes	CD	Yes	Yes	NA	Yes	NR	Yes	NA	High risk
Wutte, 2020	Yes	Yes	Yes	Yes	NR	Yes	Yes	Yes	Yes	NA	Yes	Yes	Yes	NA	Low risk
	**D1**	**D2**	**D3**	**D4**	**D5**										
**Clinical trial according Cochrane risk of bias assessment tool **															
Cengiz, 2008	Low	Low	Low	Low	Low	--	--	--	--	--	--	--	--	--	Low risk

Q: Signaling question; D: Domain; NR: Not reported; NA: Not applicable; CD: Cannot determine.

*, Any concern in these questions were considered as fetal error.

**Table 3:** Level of evidence by outcome

**Outcome**	**Sample size**	**Risk of bias**	**Imprecision**	**Inconsistency** **(I** ^2^ ** range)**	**Indirectness**	**Publication bias**	**Judgment and level of evidence**	**Level of evidence**
Overall neurological status	868	Serious	Not serious	Not serious	Not serious	Not serious	Rated down 1 point: Serious risk of bias Rated up 2 points: Large magnitude of effect Presence of plausible confounders*	**Moderate**. **⊖⊕⊕**
Neurological status based on location of injury	868	Serious	Not serious	Not serious	Not serious	Not serious	Rated down 1 point: Serious risk of bias Rated up 2 points: Large magnitude of effect Presence of plausible confounders*	**Moderate**. **⊖⊕⊕**
Neurological status based on definition of late surgery	868	Serious	Not serious	Not serious	Not serious	Not serious	Rated down 1 point: Serious risk of bias Rated up 2 points: Large magnitude of effect Presence of plausible confounders**	**Moderate**. **⊖⊕⊕**
Neurological status based on severity of injury	640	Serious	Not serious	Not serious	Not serious	Not serious	Rated down 1 point: Serious risk of bias Rated up 2 points: Large magnitude of effect Presence of plausible confounders*	**Moderate**. **⊖⊕⊕**

*, Since most of the studies had assessed cervical spinal cord patients, the authors decided that there are possible plausible confounders, which increase the confidence of findings.

**, Since late group compromise surgery after 12 hours (rather than 24 hours) in the most included studies, the authors decided that there are possible plausible confounders, which increase the confidence of findings.

## 5. Conclusion:

The present meta-analysis, performed on 15 cohorts and one clinical trial, demonstrated that ultra-early decompression surgery within the first 12 hours following SCI in patients with an AIS A injury and/or a cervical SCI may be more effective than surgeries performed after the first 12 hours following SCI in promoting neurological recovery. The efficacy of the ultra-early intervention in lumbar and thoracic injuries or less severe injuries (AIS B to D) was not proven in the present study; therefore, further clinical trials are needed in this regard.

## 6. Declarations:

### 6.1 Acknowledgments

None.

### 6.2 Authors’ contributions

Study Design: MY, RJK

Data gathering: MY, BH, RKO, AMN

Analysis: MY

Interpretation of results: RJL, RK, RKO

Drafting: MY, AMN

Critically revised: All authors

### 6.3 Funding and supports

None

### 6.4 Conflict of Interest

There are no conflicts of interest
